# Synthesis, crystal structure and Hirshfeld surface analysis of *N*-(6-acetyl-1-nitro­naphthalen-2-yl)acetamide

**DOI:** 10.1107/S2056989024001609

**Published:** 2024-03-06

**Authors:** Xin-Wei Shi, Shao-Jun Zheng, Qiang-Qiang Lu, Gen Li, Ya-fu Zhou

**Affiliations:** a Xi’an Botanical Garden of Shaanxi Province (Institute of Botany of Shaanxi Province), Shaanxi Engineering Research Centre for Conservation and Utilization of Botanical Resources, Xi’an 710061, People’s Republic of China; b School of Environmental and Chemical Engineering, Jiangsu University of Science and Technology, Zhenjiang 212003, Jiangsu, People’s Republic of China; Venezuelan Institute of Scientific Research, Venezuela

**Keywords:** crystal structure, naphthalene ring, hydrogen bonding, Hirshfeld surface analysis

## Abstract

The title compound, C_14_H_12_N_2_O_4_, was obtained from 2-acetyl-6-amino­naphthalene through two-step reactions of acetyl­ation and nitration. The mol­ecule comprises the naphthalene ring system consisting of functional systems bearing a acetyl group (C-2), a nitro group (C-5), and an acetyl­amino group (C-6). In the crystal, the mol­ecules are assembled into two-dimensional sheet-like structures by inter­molecular N—H⋯O and C—H⋯O hydrogen-bonding inter­actions. Hirshfeld surface analysis illustrates that the most important contributions to the crystal packing are from O⋯H/H⋯O (43.7%), H⋯H (31.0%), and C⋯H/H⋯C (8.5%) contacts.

## Abstract 2

The title compound, C_14_H_12_N_2_O_4_, obtained from 2-acetyl-6-aminona­phthalene through two-step reactions of acetyl­ation and nitration, is a Prodane fluorescent dye. In the crystal, the mol­ecules are assembled into two-dimensional sheet-like structures by inter­molecular N—H⋯O hydrogen bonding and π–π stacking inter­actions. Hirshfeld surface analysis indicates that the most important contributions to the crystal packing are from O⋯H/H⋯O (43.7%), H⋯H (31.0%), and C⋯H/H⋯C (8.5%) contacts.

## 1. Chemical context

Organic small mol­ecules with naphthalene ring systems are attractive photonic materials due to their high photoluminescence quantum efficiency, color tunability, and size-dependent optical properties (Wang *et al.*, 2012 ▸; Yao *et al.*, 2013 ▸). Modifying the organic mol­ecular structure can tune the inter­molecular hydrogen-bonding and π–π stacking inter­actions, which influence their packing mode during self-assembly and determine the final aggregated structures. The mol­ecular stacking patterns in crystals can affect asymmetric light propagation (Yagai *et al.*, 2012 ▸; Zou *et al.*, 2018 ▸; Zhang *et al.*, 2018 ▸). 
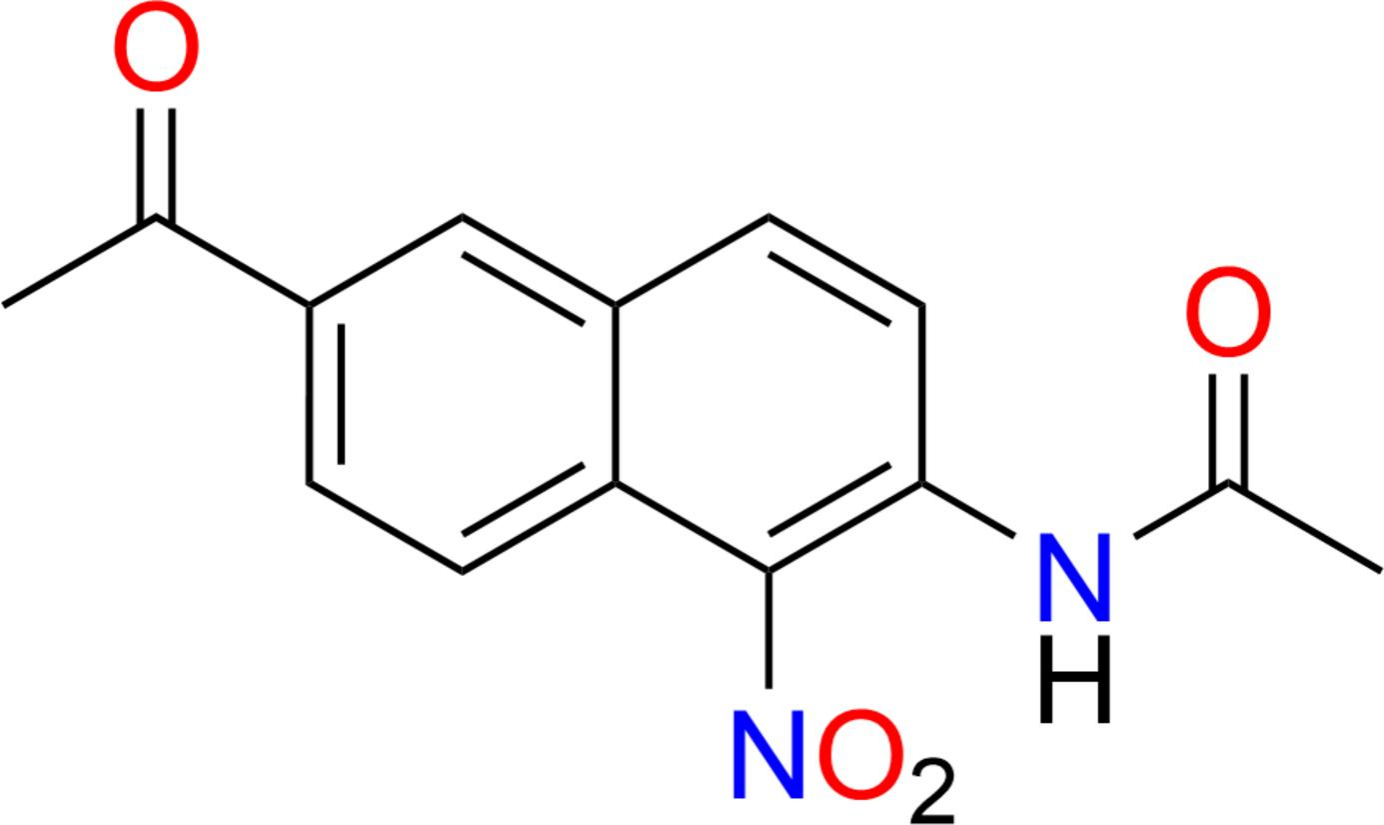


The title compound (**I**), *N*-(6-acetyl-1-nitro­naphthalen-2-yl)acetamide, obtained from 2-acetyl-6-aminona­phthalene through two-step reactions of acetyl­ation and nitration, is a Prodane fluorescent dye with red fluorescence and a large Stoke shift (Xu *et al.*, 2017 ▸). The stacking of naphthalene compounds into crystals depends on inter­molecular hydrogen bonds and π–π stacking inter­actions. The nitro group and the acetyl­amino group of the naphthalene ring system will affect inter­molecular inter­actions, making it possible to change the one- or two-dimensional stacking arrangement, which in turn affects photo-ion conduction (Eya’ane Meva *et al.*, 2012 ▸; Nguyen *et al.*, 2004 ▸).

## 2. Structural commentary

**Figure 1:**
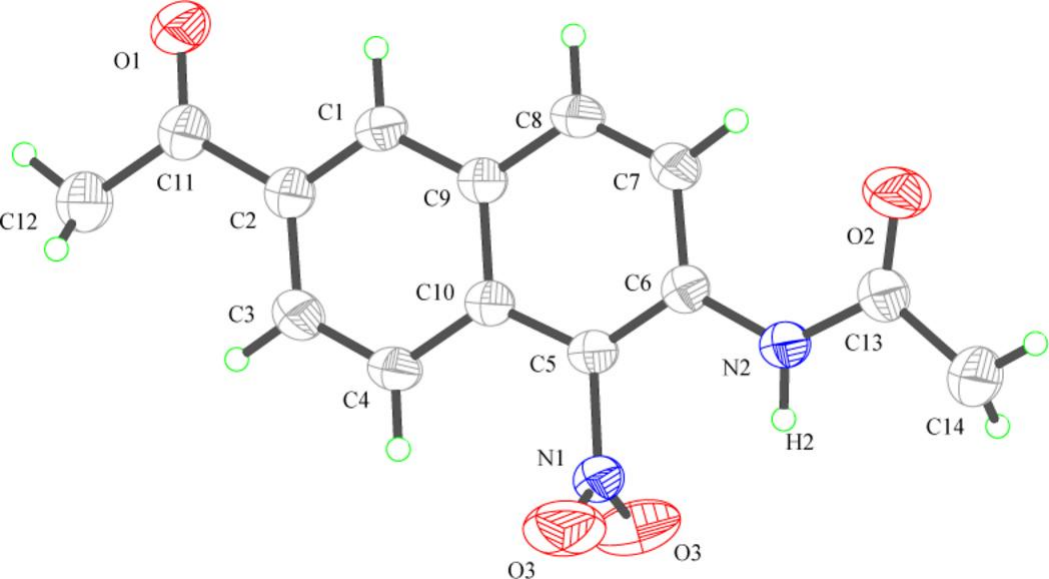
The mol­ecular structure of the title compound (I) with the atomic numbering scheme. Displacement ellipsoids are depicted at the 50% probability level.

The mol­ecular structure of the title compound (I) is shown in Fig. 1 ▸. The mol­ecules are semi-rigid and almost fully coplanar, except for the nitro oxygen atoms and methyl hydrogen atoms. Notably, compound (I) has a primary amine group on the naphthalene core, while the reactant has a secondary amine at the same position. It may have more steric repulsion with neighboring mol­ecules compared to the reactant when assembled into 2D structures. Self-assembly of naphthalene framework organic mol­ecules through π–π stacking forms 3D sheet-like structures with uniform dimensions.

In compound (I), the nitro group and acetyl­amino group are adjacent, located at positions C-5 and C-6, respectively, and the acetyl group is located at the 2-position of the naphthalene ring system. The angle between the two oxygen atoms on the nitro group located at positions C-5 is 123.93 (18)°, and the torsion angles C6—C5—N1—O3 and C10—C5—N1—O3 are −90.34 (15) and 89.66 (15)°, respectively. The angles of the acetyl group at the 2-position, O1—C11—C2 and O1—C11—C12, are 120.13 (18) and 120.52 (18)°, respectively. In addition, the dihedral angle between the nitro group and the plane through the naphthalene ring system is 89.66 (15)°.

## 3. Supra­molecular features

**Figure 2:**
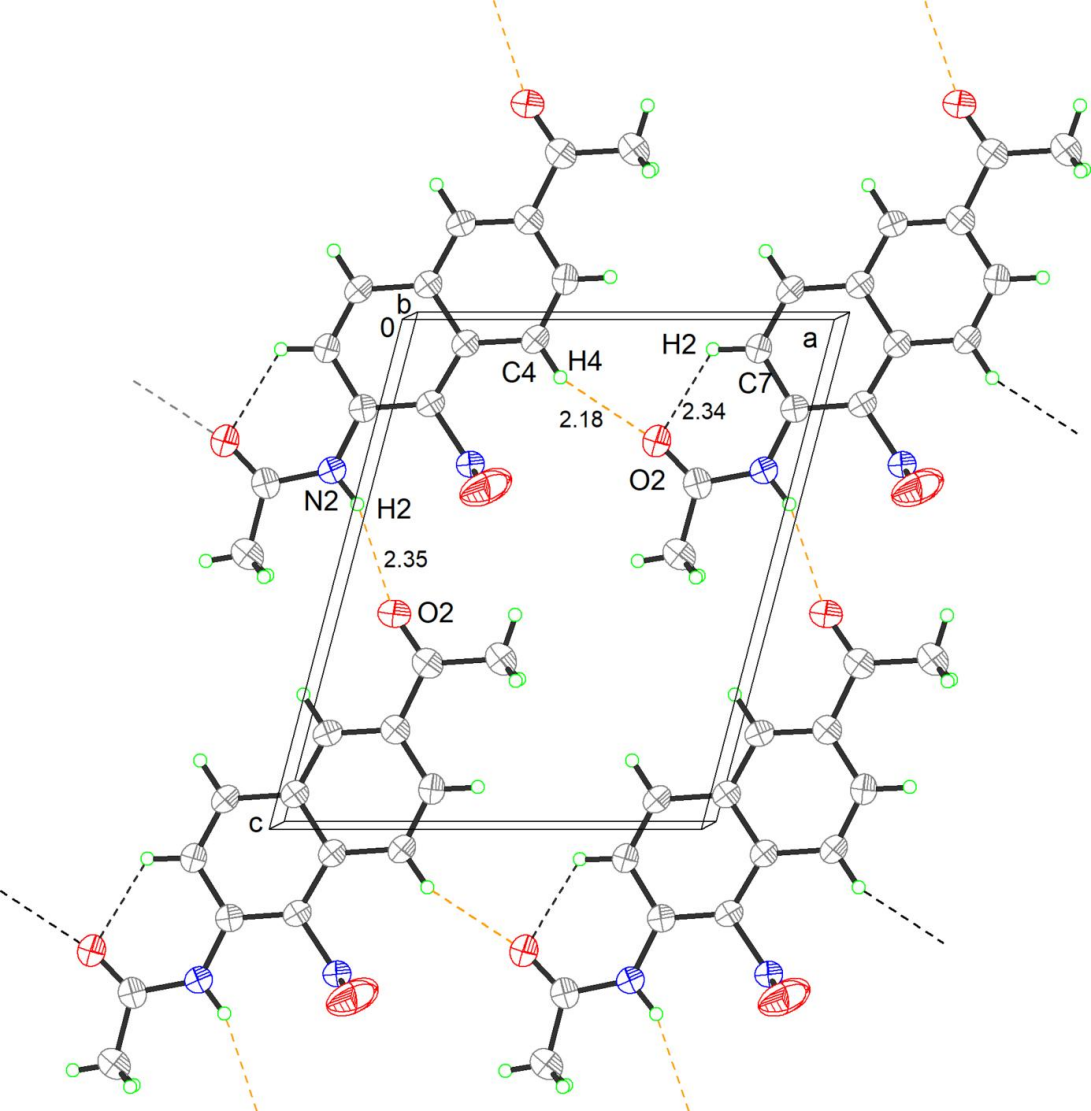
The packing of mol­ecules in the title compound (I), viewed along the *b*-axis direction (N2—H2⋯O1 hydrogen bonds are shown as orange dashed lines, C7—H7⋯O2 and C4—H4⋯O2 hydrogen bonds are shown as gray dashed lines).

In the crystal, a unit cell contains four mol­ecules, which exhibit a centrosymmetric arrangement (Fig. 2 ▸), and hydrogen bonding and π–π stacking inter­actions were responsible for the formation of the crystal structures with distinct morphologies.

The growth pattern for the title compound (I) is a 1D wire-like structure and hydrogen bonding advances the growth along the *a-*axis direction. The mol­ecules are linked *via* N2—H2⋯O1 hydrogen bonds, generating 2D layers propagating along the [010] axis direction (Table 1 ▸). Without hydrogen-bonding and other strong inter­actions between mol­ecules in adjacent layers, π–π stacking inter­actions, with centroid–centroid distcances of 3.67 Å, are the predominant driving force during self-assembly, which facilitates the crystal of the title compound growth along the [010] direction, forming a 3D structure (Meva *et al.*, 2012 ▸; Nguyen *et al.*, 2004 ▸). Weak C4—H4⋯O2 contacts are also observed.

## 4. Hirshfeld Surface analysis

**Figure 3:**
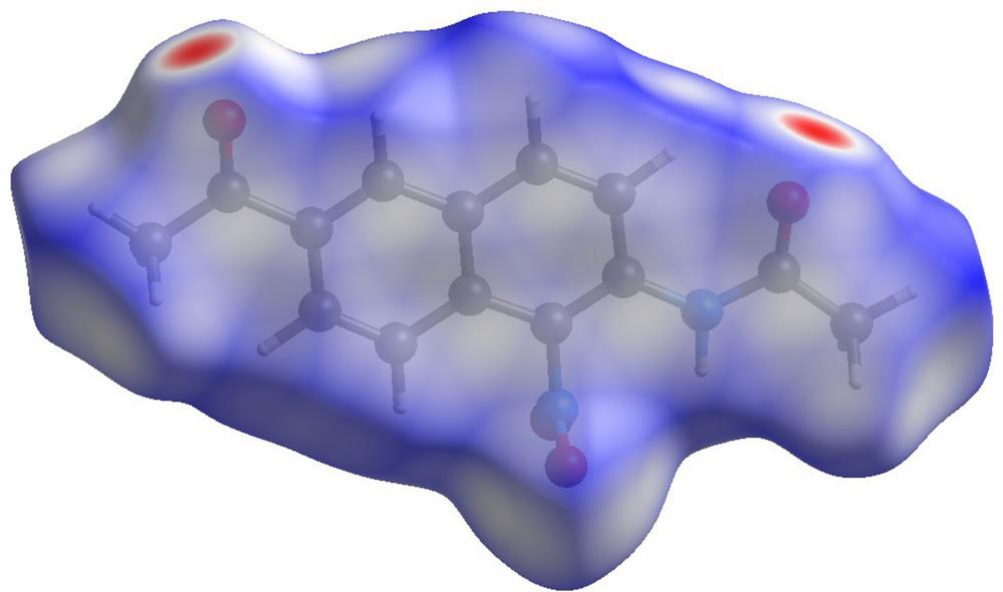
Front view of the three-dimensional Hirshfeld surface of the title compound (I) mapped over *d*_norm_.

**Figure 4:**
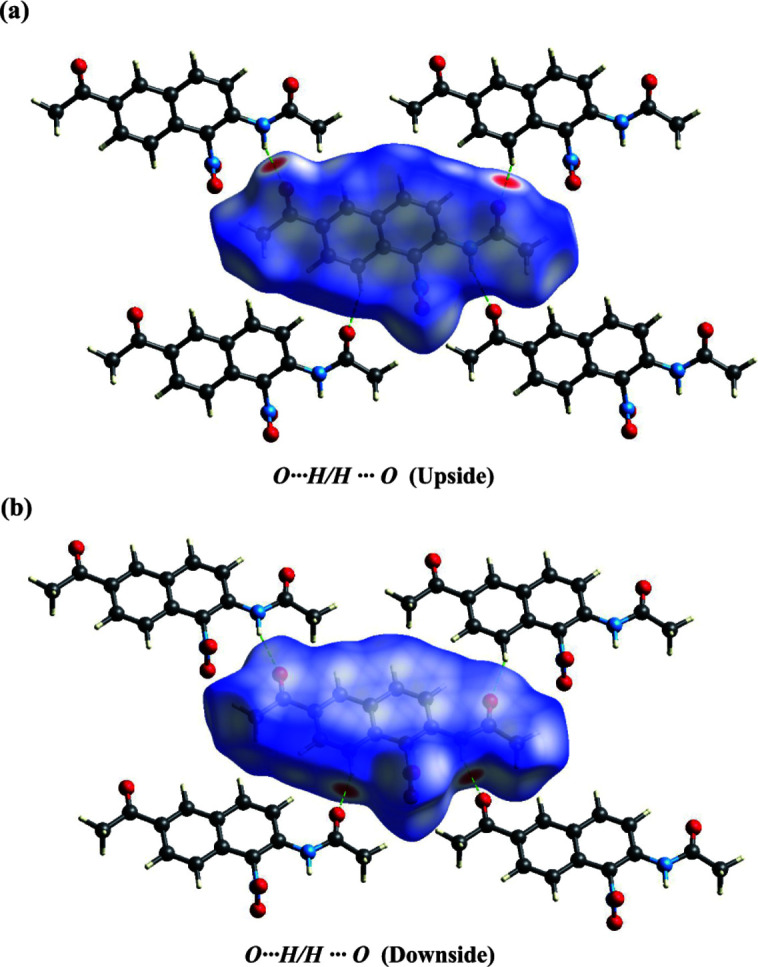
Hirshfeld surface mapped over *d*_norm_ for the title compound (I) showing: H⋯O/O⋯H (upside and downside) contacts.

A Hirshfeld surface analysis was performed and the associated fingerprint plots, which provide a 2D view of the inter­molecular inter­actions within mol­ecular crystals, were generated using *Crystal Explorer 21.5* (Spackman *et al.*, 2021 ▸), with a standard resolution of the 3D *d*_norm_ surfaces plotted over a fixed color scale of −0.1253 (red) to 1.4046 (blue) arbitrary units (Fig. 3 ▸). The N2—H2⋯O1 hydrogen bond was identified to be a crucial structure-forming inter­action within the crystal packing. The intense red spots symbolizing short contacts and negative *d*_norm_ values on the surface are related to the presence of the N2—H2⋯O1 hydrogen bonds in the crystal structure. The weak C4—H4⋯O2 contacts are indicated by faint red spots (Fig. 4 ▸).

**Figure 5:**
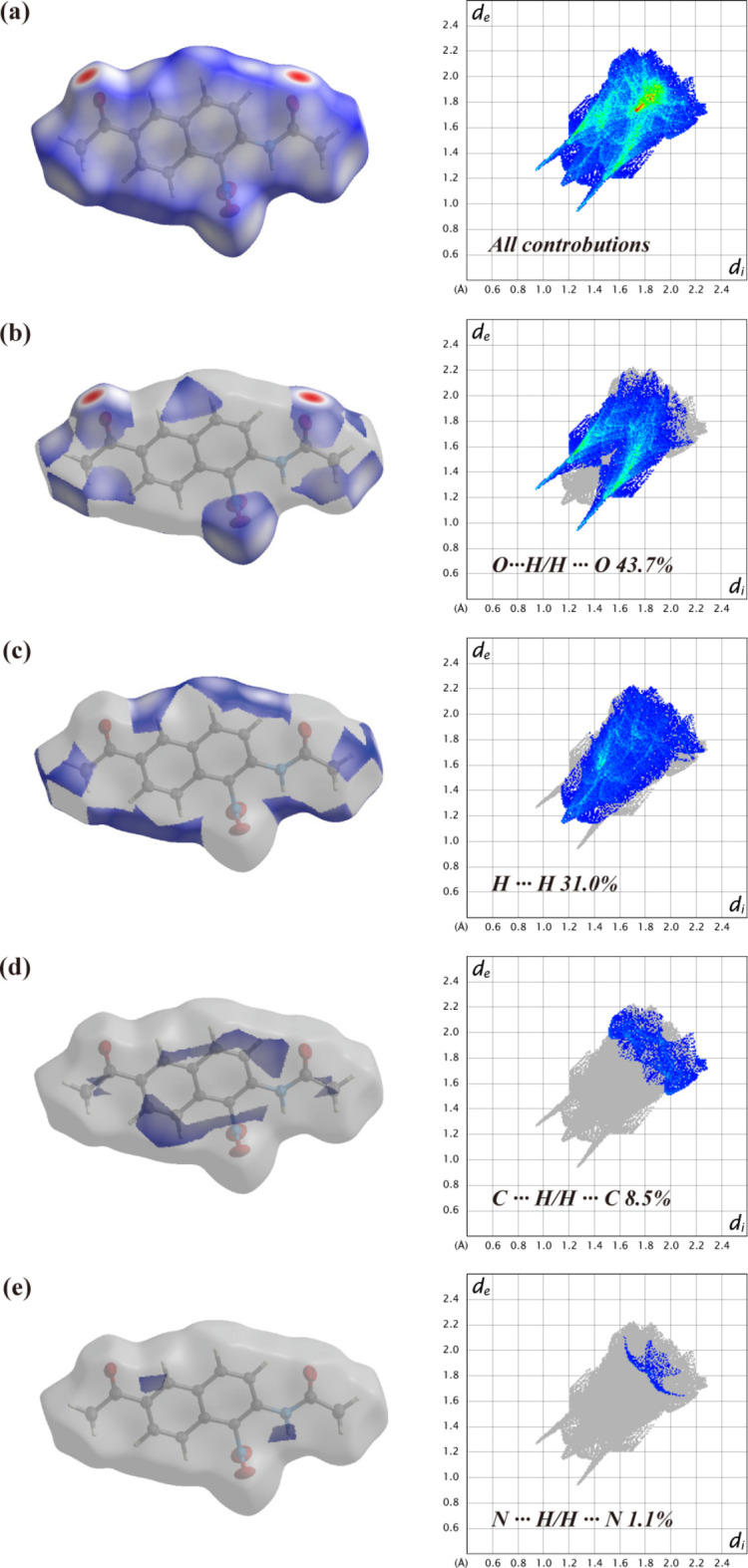
The two-dimensional fingerprint plots of the title compound (I), showing (*a*) all inter­actions, and delineated into (*b*) H⋯H, (*c*) O⋯H/H⋯O, (*d*) C⋯H/H⋯C, and (*e*) N⋯H/H⋯N inter­actions [The *d*_e_ and *d*_i_ values represent the distances (in Å) from a point on the Hirshfeld surface to the nearest atoms inside and outside the surface, respectively.]

The 2D fingerprint plots for the H⋯O/O⋯H, H⋯H, H⋯C/C⋯H, and H⋯N/N⋯H contacts are shown in Fig. 5 ▸. The most significant inter­actions are H⋯O/O⋯H, which play a defining role in the overall crystal packing, contributing 43.7%, and are located in the tip and middle region of the fingerprint plot. H⋯H inter­actions contribute 31.0%, being located in the middle region of the fingerprint plot. The contributions of the weak H⋯C/C⋯H and H⋯N/N⋯H contacts to the Hirshfeld surface are 8.5 and 1.1%, respectively.

**Figure 6:**
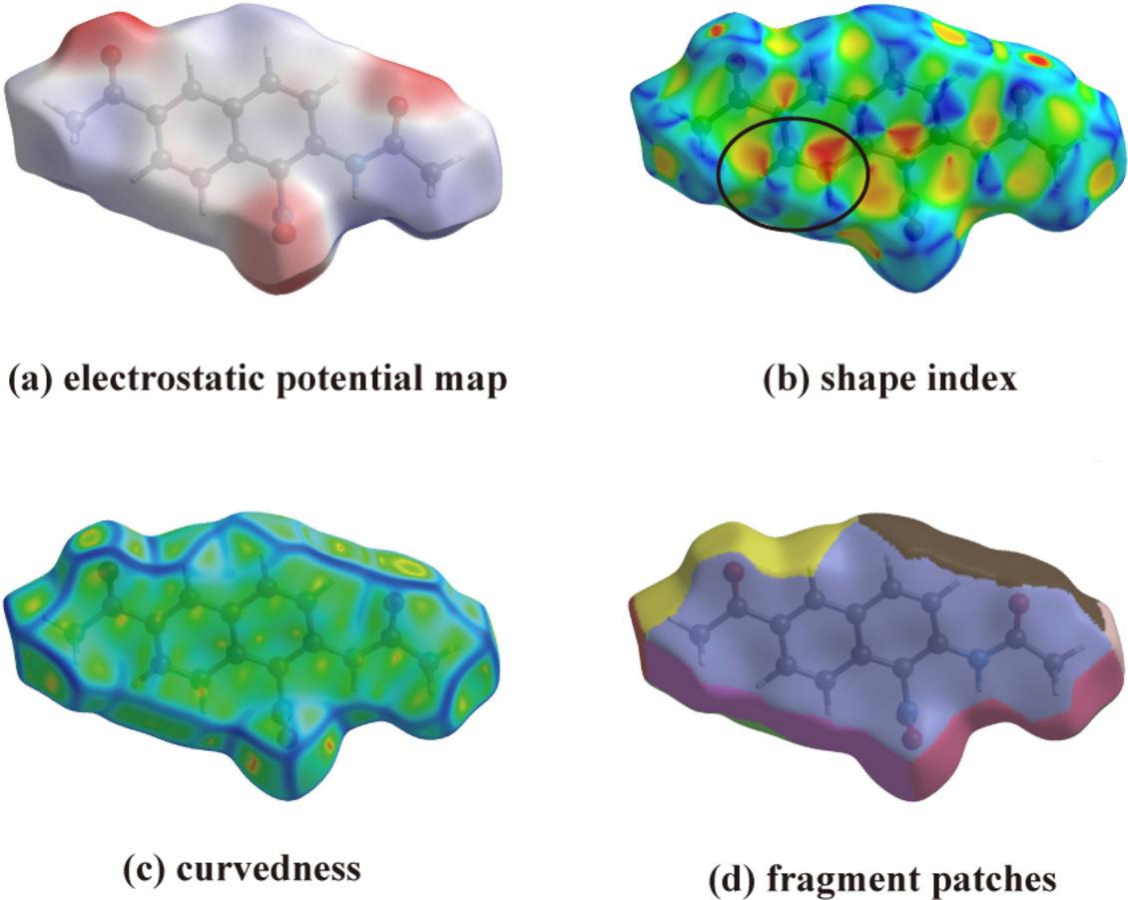
Hirshfeld surface of the title compound (I) mapped over (*a*) electrostatic potential, (*b*) shape-index, (*c*) curvedness, and (*d*) fragment patches.

Shape-index and curvedness are the metrics that describe the local shape in terms of principal curvatures, representing the surface properties of the crystal mol­ecule to determine their arrangements. The Hirshfeld surface mapped over electrostatic potential, shape-index, curvedness and fragment patches is shown in Fig. 6 ▸. The electrostatic potential map (Fig. 6 ▸ *a*) highlights the electronegative (red) and electropositive (blue) regions in the mol­ecule. The mol­ecule shows red colored regions near the oxygen atom (O1), indicating the electronegative spots (Akhileshwari *et al.*, 2021 ▸). The pattern of red and blue triangles on the shape-index map (Fig. 6 ▸ *b*) shows feature characteristic of π–π inter­actions. As the mol­ecule shows flat regions on the curvedness map (Fig. 6 ▸ *c*), it is evident that the title mol­ecule is arranged in planar stacking (Spackman & Jayatilaka, 2009 ▸). The fragment patches (Fig. 6 ▸ *d*) illustrates the coordination number of the corresponding atoms in the compound.

## 5. Synthesis and crystallization

1.0 g of 2-acetyl-6-aminona­phthalene were dissolved in 35 ml of Ac_2_O, stirred for 10 minutes, and 30 ml of CH_3_COOH were added, followed by the slow addition of 6.5 ml of concentrated HNO_3_ under ice-bath conditions for 3 h at room temperature. When the reaction was complete, it is extracted with CH_2_Cl_2_ three times, the organic phase was combined, the positive silica gel column was passed under normal pressure after spinning (eluent CH_2_Cl_2_:ethyl acetate, 10:1). The eluent containing the product components was collected and the light-yellow solid was concentrated. It was dissolved in methanol and placed in a refrigerator at 277 to cultivate light-yellow transparent square crystals (Xu *et al.*, 2017 ▸). The MeOH was dissolved and red transparent square crystals suitable for X-ray diffraction were were obtained at 277 K in the refrigerator.

## 6. Refinement

Crystal data, data collection and structure refinement details are summarized in Table 2 ▸. H atoms were positioned geometrically (C—H = 0.93–0.95 Å) and allowed to ride on their parent atoms, with *U*_iso_(H) =1.2 *U*_eq_(C) or 1.5*U*_eq_(C-meth­yl).

## Supplementary Material

Crystal structure: contains datablock(s) I. DOI: 10.1107/S2056989024001609/zn2035sup1.cif

Structure factors: contains datablock(s) I. DOI: 10.1107/S2056989024001609/zn2035Isup3.hkl

Supporting information file. DOI: 10.1107/S2056989024001609/zn2035Isup3.cml

CCDC reference: 2333518

Additional supporting information:  crystallographic information; 3D view; checkCIF report

## Figures and Tables

**Table 1:** Hydrogen-bond geometry (Å, °)

*D*—H⋯*A*	*D*—H	H⋯*A*	*D*⋯*A*	*D*—H⋯*A*
N2—H2⋯O1^i^	0.86	2.35	3.177 (2)	161
C7—H7⋯O2	0.93	2.18	2.792 (2)	123
C4—H4⋯O2^ii^	0.93	2.34	3.219 (2)	157

Symmetry codes: (i) 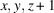 ; (ii) 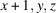 .

**Table 2:** Experimental details

Crystal data	
Chemical formula	C_14_H_12_N_2_O_4_
*M*_r_	272.26
Crystal system, space group	Monoclinic, *P*2_1_/*m*
Temperature (K)	293
*a*, *b*, *c* (Å)	8.7649 (14), 6.8899 (11), 10.6868 (18)
β (°)	104.676 (4)
*V* (Å^3^)	624.31 (18)
*Z*	2
Radiation type	Mo *K*α
μ (mm^−1^)	0.11
Crystal size (mm)	0.22 × 0.20 × 0.18

Data collection	
Diffractometer	Bruker CCD
Absorption correction	Multi-scan (*SADABS*; Krause *et al.*, 2015 ▸)
No. of measured, independent and observed [*I* > 2σ(*I*)] reflections	4087, 1229, 1079
*R*_int_	0.016
(sin θ/λ)_max_ (Å^−1^)	0.599

Refinement	
*R*[*F* ^2^ > 2σ(*F* ^2^)], *wR*(*F* ^2^), *S*	0.043, 0.124, 1.07
No. of reflections	1229
No. of parameters	118
H-atom treatment	H-atom parameters constrained
Δρ_max_, Δρ_min_ (e Å^−3^)	0.21, −0.29

Computer programs: *SMART* and *SAINT* (Bruker, 2002 ▸), *SHELXT2014/7* (Sheldrick, 2015*a* ▸), *SHELXL2014/7* (Sheldrick, 2015*b* ▸), *SHELXTL* (Sheldrick, 2008 ▸) and *publCIF* (Westrip, 2010 ▸).

## supplementary crystallographic information

### Crystal data

**Table:**

C_14_H_12_N_2_O_4_	*F*(000) = 284
*M**_r_* = 272.26	*D*_x_ = 1.448 Mg m^−^^3^
Monoclinic, *P*2_1_/*m*	Mo *K*α radiation, λ = 0.71073 Å
*a* = 8.7649 (14) Å	Cell parameters from 1846 reflections
*b* = 6.8899 (11) Å	θ = 2.4–25.0°
*c* = 10.6868 (18) Å	µ = 0.11 mm^−^^1^
β = 104.676 (4)°	*T* = 293 K
*V* = 624.31 (18) Å^3^	Block, red
*Z* = 2	0.22 × 0.20 × 0.18 mm

### Data collection

**Table:**

Bruker CCD diffractometer	1079 reflections with *I* > 2σ(*I*)
phi and ω scans	*R*_int_ = 0.016
Absorption correction: multi-scan (SADABS; Krause *et al.*, 2015)	θ_max_ = 25.2°, θ_min_ = 2.0°
	*h* = −9→10
4087 measured reflections	*k* = −8→7
1229 independent reflections	*l* = −12→12

### Refinement

**Table:**

Refinement on *F*^2^	0 restraints
Least-squares matrix: full	Hydrogen site location: mixed
*R*[*F*^2^ > 2σ(*F*^2^)] = 0.043	H-atom parameters constrained
*wR*(*F*^2^) = 0.124	*w* = 1/[σ^2^(*F*_o_^2^) + (0.0709*P*)^2^ + 0.1248*P*] where *P* = (*F*_o_^2^ + 2*F*_c_^2^)/3
*S* = 1.07	(Δ/σ)_max_ < 0.001
1229 reflections	Δρ_max_ = 0.21 e Å^−^^3^
118 parameters	Δρ_min_ = −0.29 e Å^−^^3^

### Special details

**Table:**

Geometry. All esds (except the esd in the dihedral angle between two l.s. planes) are estimated using the full covariance matrix. The cell esds are taken into account individually in the estimation of esds in distances, angles and torsion angles; correlations between esds in cell parameters are only used when they are defined by crystal symmetry. An approximate (isotropic) treatment of cell esds is used for estimating esds involving l.s. planes.

### Fractional atomic coordinates and isotropic or equivalent isotropic displacement parameters (Å^2^)

**Table:**

	*x*	*y*	*z*	*U*_iso_*/*U*_eq_	
C1	0.0705 (2)	0.7500	−0.18378 (17)	0.0351 (4)	
H1	−0.0097	0.7500	−0.2598	0.042*	
C2	0.2243 (2)	0.7500	−0.19094 (18)	0.0376 (5)	
C3	0.3449 (2)	0.7500	−0.07463 (19)	0.0438 (5)	
H3	0.4497	0.7500	−0.0786	0.053*	
C4	0.3117 (2)	0.7500	0.04290 (19)	0.0416 (5)	
H4	0.3934	0.7500	0.1179	0.050*	
C5	0.1084 (2)	0.7500	0.16916 (16)	0.0319 (4)	
C6	−0.0450 (2)	0.7500	0.17851 (17)	0.0323 (4)	
C7	−0.1648 (2)	0.7500	0.06065 (18)	0.0384 (5)	
H7	−0.2703	0.7500	0.0626	0.046*	
C8	−0.1267 (2)	0.7500	−0.05469 (18)	0.0378 (5)	
H8	−0.2076	0.7500	−0.1303	0.045*	
C9	0.0307 (2)	0.7500	−0.06439 (17)	0.0321 (4)	
C10	0.1533 (2)	0.7500	0.05139 (17)	0.0320 (4)	
C11	0.2589 (2)	0.7500	−0.32103 (19)	0.0423 (5)	
C12	0.4260 (3)	0.7500	−0.3295 (2)	0.0620 (7)	
H12A	0.4321	0.7500	−0.4154	0.074*	
H12B	0.4769	0.6362	−0.2882	0.074*	
C13	−0.2245 (2)	0.7500	0.3252 (2)	0.0440 (5)	
C14	−0.2240 (3)	0.7500	0.4642 (2)	0.0561 (6)	
H14A	−0.3152	0.7500	0.4775	0.067*	
H14B	−0.1689	0.8561	0.5077	0.067*	
N1	0.23711 (18)	0.7500	0.28778 (14)	0.0398 (4)	
N2	−0.07930 (18)	0.7500	0.29967 (15)	0.0389 (4)	
H2	0.0005	0.7500	0.3659	0.047*	
O1	0.15177 (18)	0.7500	−0.41834 (14)	0.0603 (5)	
O2	−0.34453 (19)	0.7500	0.24224 (16)	0.0925 (8)	
O3	0.28721 (15)	0.5958 (2)	0.33316 (11)	0.0761 (5)	

### Atomic displacement parameters (Å^2^)

**Table:**

	*U*^11^	*U*^22^	*U*^33^	*U*^12^	*U*^13^	*U*^23^
C1	0.0347 (10)	0.0390 (10)	0.0289 (9)	0.000	0.0032 (7)	0.000
C2	0.0365 (10)	0.0409 (10)	0.0359 (10)	0.000	0.0099 (8)	0.000
C3	0.0297 (9)	0.0613 (13)	0.0407 (11)	0.000	0.0091 (8)	0.000
C4	0.0291 (9)	0.0583 (13)	0.0340 (10)	0.000	0.0020 (7)	0.000
C5	0.0314 (9)	0.0328 (9)	0.0292 (9)	0.000	0.0032 (7)	0.000
C6	0.0333 (9)	0.0313 (9)	0.0321 (9)	0.000	0.0079 (7)	0.000
C7	0.0284 (9)	0.0498 (11)	0.0363 (10)	0.000	0.0070 (8)	0.000
C8	0.0296 (9)	0.0474 (11)	0.0324 (10)	0.000	0.0009 (7)	0.000
C9	0.0318 (9)	0.0313 (9)	0.0316 (10)	0.000	0.0052 (7)	0.000
C10	0.0309 (9)	0.0319 (9)	0.0315 (9)	0.000	0.0049 (7)	0.000
C11	0.0427 (11)	0.0474 (12)	0.0386 (11)	0.000	0.0135 (9)	0.000
C12	0.0453 (12)	0.100 (2)	0.0449 (12)	0.000	0.0190 (10)	0.000
C13	0.0358 (10)	0.0567 (13)	0.0410 (11)	0.000	0.0124 (9)	0.000
C14	0.0490 (12)	0.0812 (17)	0.0417 (12)	0.000	0.0182 (10)	0.000
N1	0.0329 (8)	0.0573 (11)	0.0288 (8)	0.000	0.0068 (7)	0.000
N2	0.0319 (8)	0.0539 (10)	0.0301 (8)	0.000	0.0062 (6)	0.000
O1	0.0463 (9)	0.1019 (14)	0.0327 (8)	0.000	0.0100 (7)	0.000
O2	0.0324 (8)	0.201 (3)	0.0432 (9)	0.000	0.0078 (7)	0.000
O3	0.0803 (9)	0.0778 (9)	0.0545 (7)	0.0232 (7)	−0.0123 (6)	0.0131 (6)

### Geometric parameters (Å, º)

**Table:**

C1—C2	1.370 (3)	C8—C9	1.410 (3)
C1—C9	1.405 (3)	C8—H8	0.9300
C1—H1	0.9300	C9—C10	1.418 (2)
C2—C3	1.413 (3)	C11—O1	1.212 (2)
C2—C11	1.496 (3)	C11—C12	1.490 (3)
C3—C4	1.359 (3)	C12—H12A	0.9328
C3—H3	0.9300	C12—H12B	0.9534
C4—C10	1.414 (3)	C13—O2	1.192 (3)
C4—H4	0.9300	C13—N2	1.367 (2)
C5—C6	1.374 (3)	C13—C14	1.484 (3)
C5—C10	1.410 (3)	C14—H14A	0.8470
C5—N1	1.468 (2)	C14—H14B	0.9329
C6—N2	1.402 (2)	N1—O3	1.2038 (14)
C6—C7	1.421 (3)	N1—O3^i^	1.2038 (14)
C7—C8	1.356 (3)	N2—H2	0.8600
C7—H7	0.9300		
			
C2—C1—C9	121.65 (17)	C1—C9—C8	122.63 (17)
C2—C1—H1	119.2	C1—C9—C10	119.02 (17)
C9—C1—H1	119.2	C8—C9—C10	118.35 (17)
C1—C2—C3	118.58 (17)	C5—C10—C4	123.87 (17)
C1—C2—C11	119.07 (18)	C5—C10—C9	117.26 (16)
C3—C2—C11	122.34 (17)	C4—C10—C9	118.87 (17)
C4—C3—C2	121.69 (18)	O1—C11—C12	120.52 (18)
C4—C3—H3	119.2	O1—C11—C2	120.13 (18)
C2—C3—H3	119.2	C12—C11—C2	119.35 (18)
C3—C4—C10	120.19 (17)	C11—C12—H12A	111.2
C3—C4—H4	119.9	C11—C12—H12B	108.9
C10—C4—H4	119.9	H12A—C12—H12B	108.6
C6—C5—C10	124.35 (16)	O2—C13—N2	122.84 (19)
C6—C5—N1	119.30 (16)	O2—C13—C14	121.53 (19)
C10—C5—N1	116.35 (15)	N2—C13—C14	115.63 (17)
C5—C6—N2	120.69 (16)	C13—C14—H14A	113.9
C5—C6—C7	116.93 (16)	C13—C14—H14B	111.7
N2—C6—C7	122.38 (16)	H14A—C14—H14B	107.9
C8—C7—C6	120.58 (17)	O3—N1—O3^i^	123.93 (18)
C8—C7—H7	119.7	O3—N1—C5	118.03 (9)
C6—C7—H7	119.7	O3^i^—N1—C5	118.03 (9)
C7—C8—C9	122.54 (17)	C13—N2—C6	127.79 (16)
C7—C8—H8	118.7	C13—N2—H2	116.1
C9—C8—H8	118.7	C6—N2—H2	116.1
			
C9—C1—C2—C3	0.000 (1)	N1—C5—C10—C9	180.000 (1)
C9—C1—C2—C11	180.000 (1)	C3—C4—C10—C5	180.000 (1)
C1—C2—C3—C4	0.000 (1)	C3—C4—C10—C9	0.0
C11—C2—C3—C4	180.000 (1)	C1—C9—C10—C5	180.000 (1)
C2—C3—C4—C10	0.000 (1)	C8—C9—C10—C5	0.0
C10—C5—C6—N2	180.000 (1)	C1—C9—C10—C4	0.000 (1)
N1—C5—C6—N2	0.000 (1)	C8—C9—C10—C4	180.0
C10—C5—C6—C7	0.000 (1)	C1—C2—C11—O1	0.000 (1)
N1—C5—C6—C7	180.000 (1)	C3—C2—C11—O1	180.000 (1)
C5—C6—C7—C8	0.000 (1)	C1—C2—C11—C12	180.000 (1)
N2—C6—C7—C8	180.000 (1)	C3—C2—C11—C12	0.000 (1)
C6—C7—C8—C9	0.000 (1)	C6—C5—N1—O3	90.34 (15)
C2—C1—C9—C8	180.000 (1)	C10—C5—N1—O3	−89.66 (15)
C2—C1—C9—C10	0.000 (1)	C6—C5—N1—O3^i^	−90.34 (15)
C7—C8—C9—C1	180.000 (1)	C10—C5—N1—O3^i^	89.66 (15)
C7—C8—C9—C10	0.000 (1)	O2—C13—N2—C6	0.000 (1)
C6—C5—C10—C4	180.000 (1)	C14—C13—N2—C6	180.000 (1)
N1—C5—C10—C4	0.000 (1)	C5—C6—N2—C13	180.000 (1)
C6—C5—C10—C9	0.000 (1)	C7—C6—N2—C13	0.000 (1)

Symmetry code: (i) *x*, −*y*+3/2, *z*.

### Hydrogen-bond geometry (Å, º)

**Table:**

*D*—H···*A*	*D*—H	H···*A*	*D*···*A*	*D*—H···*A*
N2—H2···O1^ii^	0.86	2.35	3.177 (2)	161
C7—H7···O2	0.93	2.18	2.792 (2)	123
C4—H4···O2^iii^	0.93	2.34	3.219 (2)	157

Symmetry codes: (ii) *x*, *y*, *z*+1; (iii) *x*+1, *y*, *z*.
